# Surgical and oncological implications of the presence of hepatic artery anatomical variations in patients undergoing pancreaticoduodenectomy: a single center experience

**DOI:** 10.1007/s13304-025-02079-3

**Published:** 2025-01-29

**Authors:** Laura Alberici, Claudio Ricci, Vincenzo D’Ambra, Carlo Ingaldi, Margherita Minghetti, Carlo Mazzucchelli, Riccardo Casadei

**Affiliations:** 1 Pancreatic and Endocrine Surgical Unit, IRCCS Azienda Ospedaliero-Universitaria Di Bologna, Bologna, Italy; 2 Department of Internal Medicine and Surgery (DIMEC), Alma Mater Studiorum, University of Bologna, Bologna, Italy

**Keywords:** Aberrant hepatic artery, Vascular anatomy, PDAC, Pancreaticoduodenectomy, Arterial margin, Local recurrence

## Abstract

The presence of an aberrant right hepatic artery (a-RHA) could influence the oncological and postoperative outcomes after pancreaticoduodenectomy (PD). A comparative study was conducted, including patients who underwent PD with a-RHA or with normal RHA anatomy. The primary endpoints were R1 resection in all margins (pancreatic, anterior, posterior, superior mesenteric artery, and portal groove), overall survival (OS), and disease-free survival (DFS). The secondary endpoints were morbidity, mortality, biliary leak (BL), postoperative pancreatic fistula (POPF), delayed gastric emptying (DGE), post pancreatectomy hemorrhage (PPH), length of stay (LOS), and operative time (OT). The selection bias was measured using the *d*-value and limited using entropy balancing analysis. Patients with arterial variants were 84 (37.5%), 39 (17.4%) of whom had a-RHA from SMA. The patients with a-RHA were more frequently symptomatic (*d* = 0.652), often affected by PDAC (*d* = 0.369), and judged borderline resectable (*d* = 0.588). Neoadjuvant therapy was more frequently proposed for patients with a-RHA (*d* = 0.465). The patients with a-RHA often had the Wirsung dilated compared to those without a-RHA (*d* = 0.336). After bias correction, the R1 resection rate on the superior mesenteric artery (SMA) margin was higher in the a-RHA group than in patients with normal RHA anatomy (OR 2.3; 1.1–5.2; *P* = 0.045). OS, DFS, morbidity, mortality, biliary leak (BL), postoperative pancreatic fistula (POPF), delayed gastric emptying (DGE), post pancreatectomy hemorrhage (PPH), length of stay (LOS), and operative time (OT) were similar in unmatched and matched population. The presence of a-RHA seems to increase the risk of R1 resection in the SMA margin.

**Supplementary Information:**

The online version contains supplementary material available at 10.1007/s13304-025-02079-3.

## Introduction

Pancreaticoduodenectomy (PD) is a technically demanding surgical intervention burdened by a non-negligible complication rate. It involves an extended demolition phase, with en-bloc resection of the gastric antrum, the duodenum, the head of the pancreas, the distal common bile duct, and the gallbladder and a subsequent complex reconstructive phase consisting of three challenging anastomoses. Beyond that, between the two phases, lymphadenectomy and a complete dissection of both retro-portal lamina and meso-pancreas must be performed being aware of possible vascular anatomical variations, first among all, the presence of a right hepatic artery (RHA) arising from the superior mesenteric artery (SMA), to maintain the integrity of the hepatic artery blood supply [1]. Variations in the vascular pattern of the celiac axis (CA) and its branches have been reported by several studies [2, 3], with an incidence in the general population ranging between 13 and 48% [4, 5]. The most employed classifications describing these vascular anomalies in the literature are Michels’ classification [6], based on 200 autopsies, and its more recent modification by Hiatt [7], based on 1000 angiographic analyses. Among the described ones, the most common variant is an aberrant right hepatic artery (a-RHA) arising from the SMA. This has been reported to occur in 11–21% of patients [8]. A distinction between an accessory and replaced a-RHA can be made by classifying as an accessory right hepatic artery (acc-RHA), an artery arising from the SMA that adds to a normal RHA arising from CA and as a replaced right hepatic artery (rep-RHA) an artery arising from the superior mesenteric artery (SMA) and supplying on its own most of the right lobe of the liver as well as the middle part of the common bile duct and the gallbladder, in the absence of a branch from the CA.

In the event of an injury to an a-RHA during PD, consequences may both compromise the hepatic artery blood supply and increase the risk of postoperative biliary anastomotic leak [9, 10]. The detection of such vascular anomalies can be preoperative or intraoperative. Routine preoperative CT scans can provide useful information about the existence of such anomalies enabling better preparation to manage the aberrant vessels during the intervention [9]. Surgeon’s experience and expertise should consider, on the one hand, the necessity to preserve the vascular anatomy and its anomalies in every single patient and, on the other hand, the intent to achieve complete clearance of retro pancreatic tissues since this has been proven to be an independent risk factor for disease recurrence and hence patient survival [11].

The purpose of our study was to analyze a cohort of patients undergoing PD to define if the presence of hepatic artery anatomical variations has an impact on the surgical procedures themselves (in terms of operative time, iatrogenic injury, change in surgical strategy) and on the patient’s prognosis (in terms of R0 vs. R1 resection or disease recurrence during follow up).

Previous studies have investigated oncological outcomes in patients with RHA variations undergoing PD for peri-ampullary malignancies and found that the R0/R1 resection rate was not compromised by the presence of an a-RHA [10].

However, these studies broadly considered the R status on the specimen without focusing on the specific AMS margin status. We specifically considered the impact of vascular anomalies involving the RHA origin to detect whether these could compromise the R status on the AMS margin.

## Materials and methods

The study was based on a prospectively maintained database of pancreatic resections, approved by the local ethical committee with code PANBO 064/2017/U/Oss. All procedures were performed between 2012 and 2022 at the Division of Pancreatic Surgery, IRCCS “Azienda Ospedaliero-Universitaria” of Bologna by a surgical team including at least two experienced hepatobiliary or pancreatic surgeons. All records were screened, but only those that met the following inclusion criteria were considered: (a) patients who underwent PD; (b) diagnosis of resectable or borderline resectable PDAC or other peri-ampullary malignancies; (c) availability of a preoperative high-quality CT scan performed with arterial and portal phase to identify CA, SMA, and their respective branches; (d) pathological report that distinguished all resection margins (anterior surface, superior mesenteric vein-facing surface, superior mesenteric artery-facing surface, posterior surface, and pancreatic transection surface) according to Verbeke et al. [12]. Exclusion criteria were considered the unavailability of previously reported data and the presence of metastatic disease or locally advanced disease precluding curative intent surgery. Also, patients who received arterial (acc-RHA or rep-RHA) resection and reconstruction were excluded from the study to avoid an overestimation of the R0 rate in patients with RHA origin anomalies. For included patients, the type of vascular anomaly was described according to Michels’ classification [6] and reported for descriptive purposes. Then, the included patients were divided into two groups: patients with a-RHA from SMA (acc-RHA, rep-RHA, or CHA from SMA) and patients without these anomalies. The two groups were matched, and all differences depending on confounding covariates were measured and corrected using the entropy balancing approach [13]. As confounding variables, we reported demographic, clinical, and surgical data. Of note, we defined symptomatic only the patients with jaundice, cholangitis, back, abdominal pain, steatorrhea, or new-onset diabetes. We defined as extended those resection involving neighboring organs such as the colon or the stomach. Finally, the oncological and postoperative results of the unmatched and matched cohorts were compared. The primary endpoint was the R0/R1 resection rate in the AMS margin. The secondary endpoints were the R0/R1 resection rate in the transection, anterior, posterior, and SMV margins. In accordance with the Royal College of Pathologists, the resection margin was considered R1 when cancer cells were found ≤ 1 mm from the resection line [14]. The operative time and postoperative course were also evaluated as secondary endpoints. Operative time was calculated from the time to incision to skin suture. The postoperative course was classified at discharge, according to CDC [15]. Clinically relevant postoperative pancreatic fistula (CR-POPF), post-pancreatectomy hemorrhage (PPH), and delayed gastric emptying (DGE) were defined according to the 2016 updated ISGPF [16] and ISGPS definitions [17, 18]. Length of stay (LOS) was determined from the day of surgery to the day of discharge. OS survival was calculated from the date of surgery to the last contact or death. DFS was calculated from the date of surgery to the date of recurrence demonstrated by a CT scan or MRI. It should be noted that the evaluation of OS and DFS was restricted, considering only PDAC. In those same patients, we also evaluated the site of recurrence.

### Statistical analysis

Data were reported in percentages or with mean and standard deviation (SD). Group differences were reported using the *d*-value (standardized mean difference). A *d*-value ≤ 0.2 indicates a small difference (percentage of the non-overlap population ≤ 15%); a *d*-value > 0.2 and ≤ 0.5 indicates a medium difference (percentage of the non-overlapped population > 15% but ≤ 33%); a *d* value > 0.5 and 0.8 means a large difference, indicating a percentage of non-overlap population > 33% [13]. All the endpoints were compared for the unmatched and matched populations. Hainmueller’s “entropy balance” was applied [19]. This relatively novel approach permits eliminating all confounding biases in matching two groups. In contrast to the other pre-processing methods, such as propensity score matching, entropy balancing involves a reweighting scheme that directly incorporates covariate balance into the weight function that is applied to the sample unit. In other words, entropy balancing does not eliminate the uncommon cases, but it reweights the characteristics of patients in a cohort to be similar to the comparative group. Reweighting was performed on patients with an a-RHA to generate a virtual group with identical covariate distributions of those with normal RHA anatomy. Subsequently, classical statistical analysis was carried out, introducing obtained weights. The statistical analysis was computed using STATA software (17.0 Standard Edition—StataCorp. LCC 1985-2021. College Station, TX, USA). Entropy balance was performed with “ebalance” module.

## Results

**Fig. 1:**
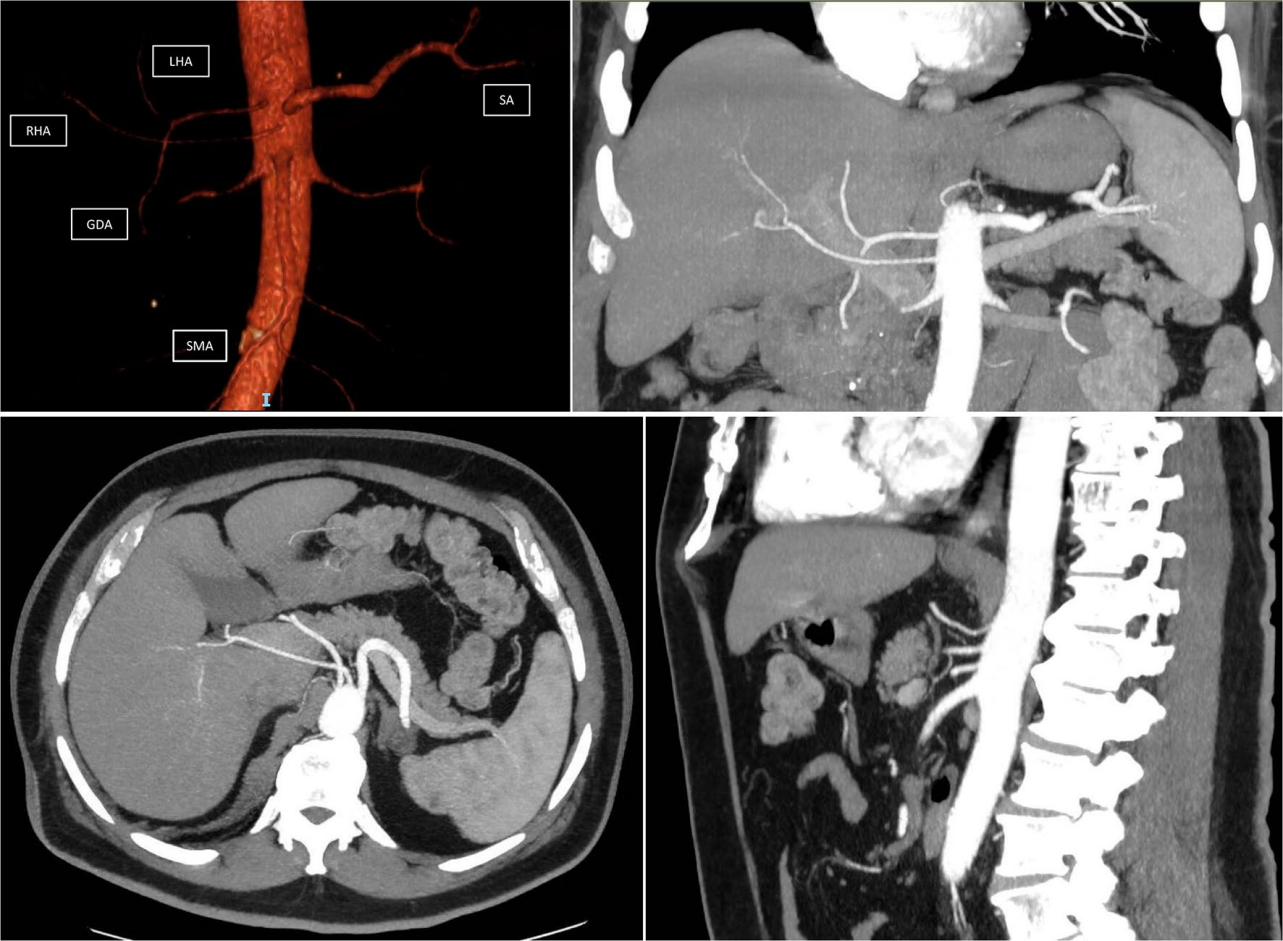
Absence of celiac trunk. The right and left hepatic arteries (RHA and LHA), the splenic artery (SA), and the left gastric artery arise separately from the aorta. The gastro-duodenal artery (GDA) originates from the left hepatic artery

**Fig. 2:**
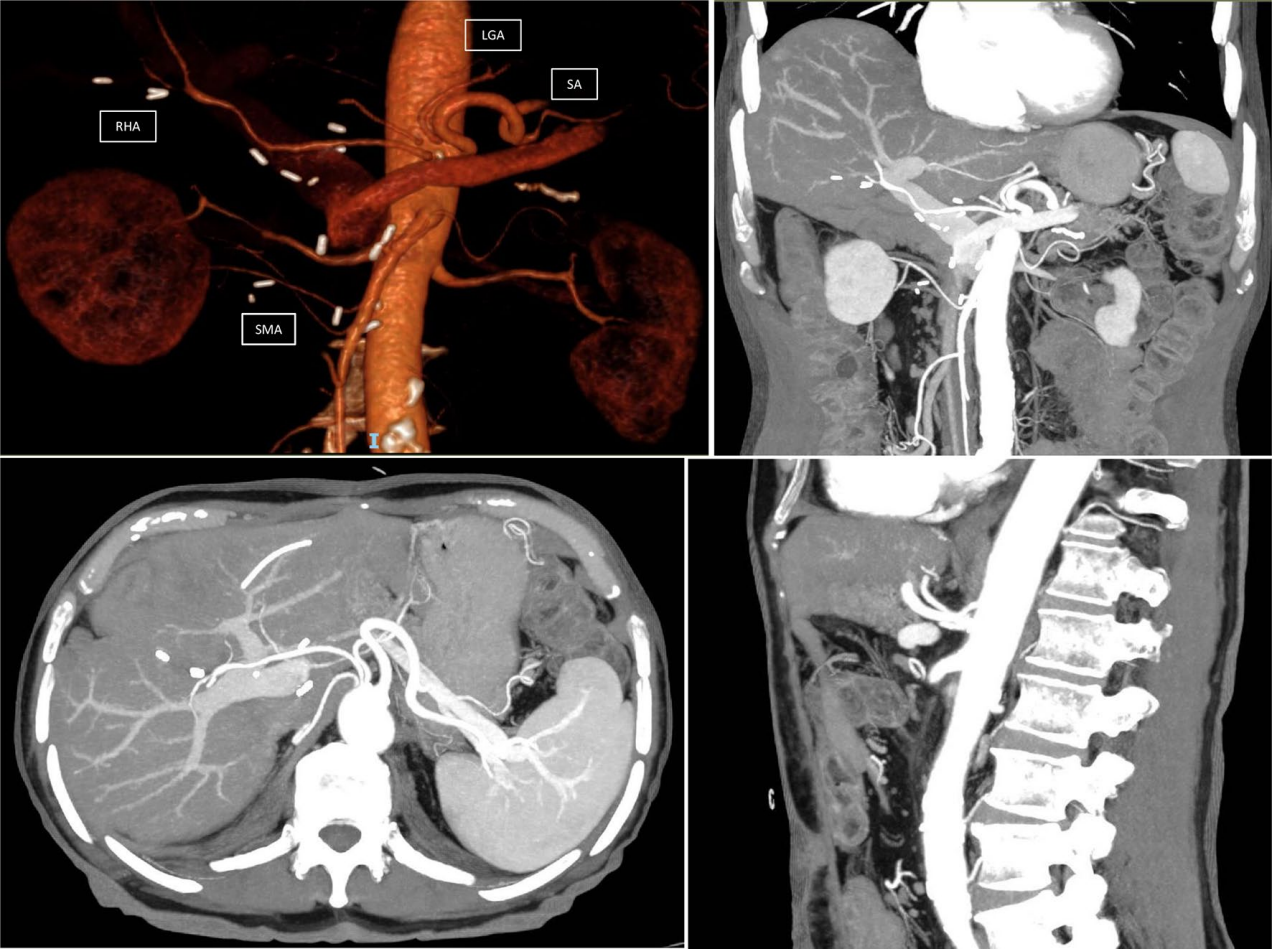
Absence of celiac trunk. The right hepatic artery (RHA), the splenic artery (SA), and the left gastric artery (LGA) arise separately from the aorta. The left hepatic artery (LHA) is a branch of the left gastric artery (LGA). A phrenic artery from the aorta is also detectable

The flowchart of the patients’ selection process is reported in Fig. 1. Patients with normal hepatic artery anatomy were 140 (62.5%). The anomalies are reported in Supplementary Table 1 according to Michels’ classification. The characteristics of the two groups are compared in Table 1. The two groups have small differences in sex (*d* = 0.175), age (*d* = 0.020), comorbidity (*d* = 0.100), pancreatic texture (*d* = 0.135), need for extended (*d* = 0.175) or vascular resection (*d* = 0.175) and type of surgeon performing pancreatic resection (*d* = 0.142). The patients with a-RHA were more frequently symptomatic (87.2 vs. 68%, *d* = 0.652), often affected by PDAC (79.5 vs. 66.5%, *d* = 0.369), and judged borderline resectable (10.3 vs. 3.8%, *d* = 0.588). Neoadjuvant therapy was more frequently proposed for patients with a-RHA (12.8 vs. 6%; *d* = 0.465). The patients with a-RHA often have the Wirsung dilated compared to those without a-RHA (48.7 vs. 34%, *d* = 0.336). After reweighting, entropy balancing disappeared all differences (*d*-values < 0.200) because the a-RHA group assumes the same characteristics as the control group. Table 2 shows the results for the unmatched and matched cohorts. Comparison between the two unmatched groups showed that patients with acc-RHA or rep-RHA have a higher risk of R1 resection in the SMA margin (35.9 vs. 18.9%; OR 2.4; *p* = 0.022). The adjusted OR obtained after reweighting was 2.3 (1.1–5.2; *p* = 0.045), confirming a higher risk in a-RHA group. No other significant differences were observed in unmatched groups for all remaining resection margins. Restricting the analysis only to the patients with PDAC, local OS, and DFS were similar in unmatched groups. (Fig. 2, panels A–D). The rate of local recurrence was slightly higher in the a-RHA group (19.3 vs 16.3%), but this difference was not statistically significant (*p* = 0.681). After reweighting, no other significant differences were observed in oncological outcomes, including local recurrence, OS, and DFS in patients with PDAC. About perioperative results, operative time was slightly longer in patients with a-RHA (MD = + 25 min, *p* = 0.039) when unmatched cohorts were compared. Major complications, CR-POPF, PPH, early PPH, DGE, LOS, and 90-day mortality were similar. After reweighting, the difference in operative time disappeared, while major complications, CR-POPF, PPH, DGE, LOS, and 90-day mortality remained similar.

**Table 1:** Characteristics of 224 patients included in the study

Parameters	No RHA from SMA (*N* = 185)	Rep RHA or Acc RHA from SMA (*N* = 39)	*d*-value	*d*-value after reweighting
Sex				
Female Male	100 (54.1) 85 (45.9)	18 (46.2) 21 (53.8)	0.175	0
Age, years	67 ± 12	70 ± 10	0.020	0.001
BMI (Kg/m^2^)	25 ± 4	25 ± 4	0	0
Comorbidity				
No One or more	50 (27) 135 (73)	12 (31) 27 (69)	0.100	0.002
Symptoms				
No Yes	60 (32) 125 (68)	5 (12.8) 34 (87.2)	0.652	0
Tumors				
PDAC Others periampullary malignancy	123 (66.5) 62 (33.5)	31 (79.5) 8 (20.5)	0.369	0
NCCN				
Resectable Borderline resectable	178 (96.2) 7 (3.8)	35 (89.7) 4 (10.3)	0.588	0
Neoadjuvant therapy				
No Yes	174 (94) 11 (6)	34 (87.2) 5 (12.8)	0.465	0
Texture of pancreatic remnant				
Soft Hard	133 (71.9) 52 (28.1)	26 (66.7) 13 (33.3)	0.135	0
Wirsung				
≤ 3 mm > 3 mm	122 (66) 63 (34)	20 (51.3) 19 (48.7)	0.336	0
Extended resection				
No Yes	166 (89.7) 19 (10.3)	36 (92.3) 3 (7.7)	0.175	0
Vascular resection				
No Yes	178 (96.2) 7 (3.8)	37 (94.9) 2 (5.1)	0.175	0
Surgeon				
Hepatic surgeon Pancreatic surgeon	15 (8.1) 170 (91.9)	4 (10.3) 35 (87.7)	0.142	0

*Rep*. replaced hepatic artery, *Acc* accessory hepatic artery, *RHA* right hepatic artery, *SMA* superior mesenteric artery, *BMI* body mass index, *PDAC* pancreatic ductal adenocarcinoma, *NCCN* National Comprehensive Cancer Network, *d-value* effect size categories: 0–0.2 small (percentage of non-overlap population < 15%); > 0.2–0.5 medium (percentage of non-overlap population < 33%); > 0.50–0.80 large (percentage of non-overlap population < 50%); over 0.8 very large (percentage of non-overlap population > 50%)

**Table 2:** Postoperative and long-term results in unbalanced and balanced groups

Postoperative and long term results	No RHA from SMA (*N* = 185)	Rep or Acc RHA from SMA (*N* = 39)	OR/MD/HR (95%CI)	*P*-value	Adj-OR/MD/HR (95%CI)	*P*-value
*Primary oncological endpoint*						
SMA margin						
R0 R1	150 (81.1) 35 (18.9)	25 (64.1) 14 (35.9)	Ref 2.4 (1.1 to 5.1)	0.022	Ref 2.3 (1.1 to 5.2)	0.045
*Secondary oncological endpoints*						
All margins						
R0 R1	106 (57.3) 79 (42.7)	18 (46.2) 21 (53.8)	Ref 1.6 (0.8 to 3.1)	0.205	Ref 1.2 (0.6 to 2.5)	0.668
PV/SMV margin						
R0 R1	144 (77.8) 41 (22.2)	31 (79.5) 8 (20.5)	Ref 0.9 (0.4 to 2.1)	0.821	Ref 0.7 (0.3 to 1.8)	0.514
Anterior margin						
R0 R1	172 (92.9) 13 (7.1)	37 (94.9) 2 (5.1)	Ref 0.7 (0.2 to 3.3)	0.668	Ref 0.7 (0.1 to 3.5)	0.670
Posterior margin						
R0 R1	143 (77.3) 42 (22.7)	26 (66.7) 13 (33.3)	Ref 1.7 (0.8 to 3.6)	0.164	Ref 1.7 (0.8 to 3.9)	0.186
Pancreatic margin						
Primary R0 Secondary R0	183 (98.9) 2 (1.1)	38 (97.4) 1 (2.6)	Ref 2.4 (0.2 to 27.2)	0.478	Ref 0.6 (0.1 to 7.7)	0.684
Local recurrence^a^						
No Yes	103 (83.7) 40 (16.3)	25 (80.7) 6 (19.3)	Ref 1.4 (0.4 to 3.4)	0.681	Ref 1.2 (0.4 to 3.6)	0.738
OS^a^	40 ± 8	35 ± 12	1.1 (0.6 to 1.9)	0.835	1.1 (0.5 to 1.9)	0.887
DFS^a^	18 ± 4	18 ± 6	0.9 (0.5 to 1.6)	0.772	0.9 (0.5 to 1.5)	0.574
*Others clinical endpoints*						
Operative time	354 ± 79	379 ± 78	+ 25 (3 to 53)	0.039	+ 21 (− 8 to 50)	0.159
CDC ≥ IIIa						
No Yes	118 (63.8) 67 (36.2)	27 (69.2) 12 (30.8)	Ref 0.8 (0.4 to 1.6)	0.518	Ref 0.7 (0.3 to 1.5)	0.331
CR-POPF						
No Yes	174 (94.1) 11 (5.9)	36 (92.3) 3 (7.7)	Ref 1.3 (0.3 to 4.9)	0.683	Ref 1.3 (0.3 to 5.2)	0.737
PPH						
No Yes	143 (77.3) 42 (22.7)	30 (76.9) 9 (23.1)	Ref 1.1 (0.4 to 2.3)	0.960	Ref 0.8 (0.3 to 1.8)	0.536
Early PPH						
No Yes	181 (97.8) 4 (2.2)	37 (94.9) 2 (5.3)	Ref 1.1 (0.4 to 13.8)	0.312	Ref 2.1 (0.3 to 14.1)	0.433
DGE						
No Yes	147 (79.5) 38 (20.5)	31 (79.5) 8 (20.5)	Ref 0.9 (0.4 to 2.3)	0.997	Ref 0.9 (0.3 to 2.2)	0.795
90-day mortality						
No Yes	172 (93) 13 (7)	36 (92.3) 3 (7.7)	Ref 1.1 (0.3 to 4.1)	0.883	Ref 1.1 (0.3 to 4.4)	0.851
LOS (days)	23 ± 17	21 ± 12	− 1.9 (− 7.7 to 3.8)	0.491	− 1.9 (− 6.7 to 2.8)	0.419

*Rep.* replaced hepatic artery, *Acc* accessory hepatic artery, *RHA* right hepatic artery, *OR* Odds ratio, *MD* mean difference, *HR* hazard ratio, *CI* confidence interval, *Adj* adjusted OR/MD/HR after entropy balancing, *SMA* superior mesenteric artery, *PV* portal vein, *SMV* superior mesenteric vein, *OS* overall survival, *DFS* disease-free survival, *CDC* Clavien-Dindo classification, *POPF* postoperative pancreatic fistula according to 2016 update International Study Group of Pancreatic Fistula (ISGPF) definition, *PPH* post-pancreatectomy hemorrhage, *DGE* delayed gastric emptying, *LOS* length of postoperative stay

^a^Restricted to pancreatic ductal adenocarcinoma

## Discussion

The present study demonstrated for the first time that the presence of an a-RHA could influence the R1 rate in the SMA margin. The occurrence of anomaly hepatic artery system is common within the population, with a prevalence of 15–25% [10]. The anatomy of RHA has attracted the attention of pancreatic surgeons, particularly for the oncological implication of a-RHA during PD. Indeed, both replaced and accessory a-RHA, arising from the SMA, cross the back surface of the head of the pancreas through the “meso-pancreas” and near to “retro-portal” lamina to join in the hepatoduodenal ligament. The term meso-pancreas was used to describe retro-pancreatic tissue, consisting of adipose tissue, nerves and lymphatic vessels, and lymph nodes extending from the posterior surface of the head and uncinate process behind the superior mesenteric vein to the right or left side of the SMA [11]. The “retro-portal margin” was described instead as the soft tissue directly adjacent to the proximal 3–4 cm of SMA [12].

**Fig. 3:**
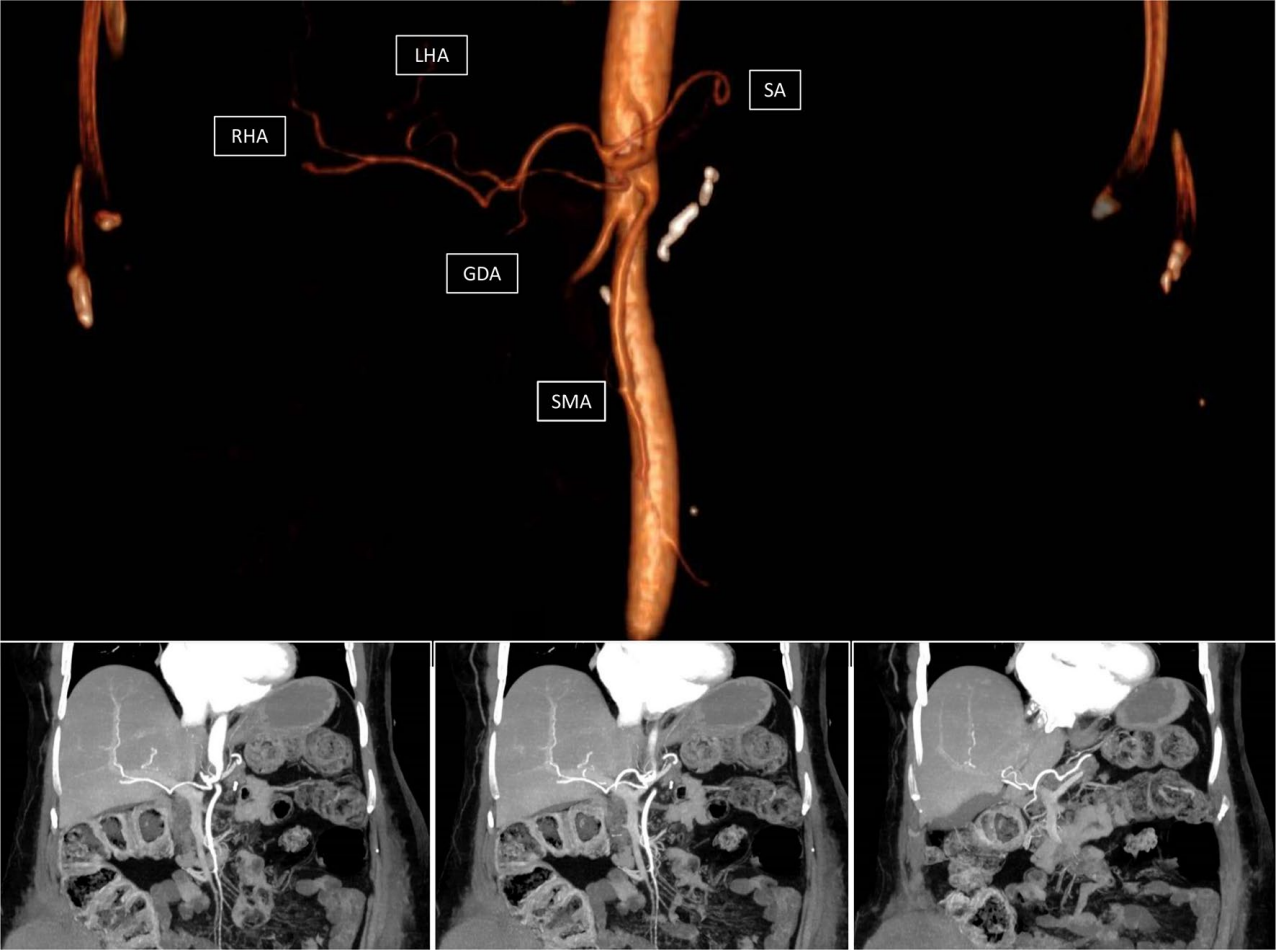
Absence of common and proper hepatic artery. The right and left hepatic arteries (RHA and LHA) arise separately from the celiac trunk. The gastro-duodenal artery (GDA) originates from the left hepatic artery (LHA)

When the R1 resection involves the SMA margin, the risk of local recurrence increases [20]. Based on these anatomical and oncological considerations, several authors have investigated the impact of a-RHA in increasing R1 resection or reducing OS in patients who underwent PD for malignancy [20–42]. These studies have controversial results, failing to demonstrate a significant increase in the R1 resection rate. However, most of the studies did not distinguish the arterial margins from other pancreatic resection lines, such as posterior or anterior margins, and this bias weakens the conclusions. In the present study, the margination of the specimen after PD was made according to the Leeds protocol [43], which distinguished all margins, including the SMA margin [44]. This approach permits the evaluation of the risk of nonradical surgery in the presence of a-RHA, avoiding the bias mentioned above (Fig. 3).

**Fig. 4:**
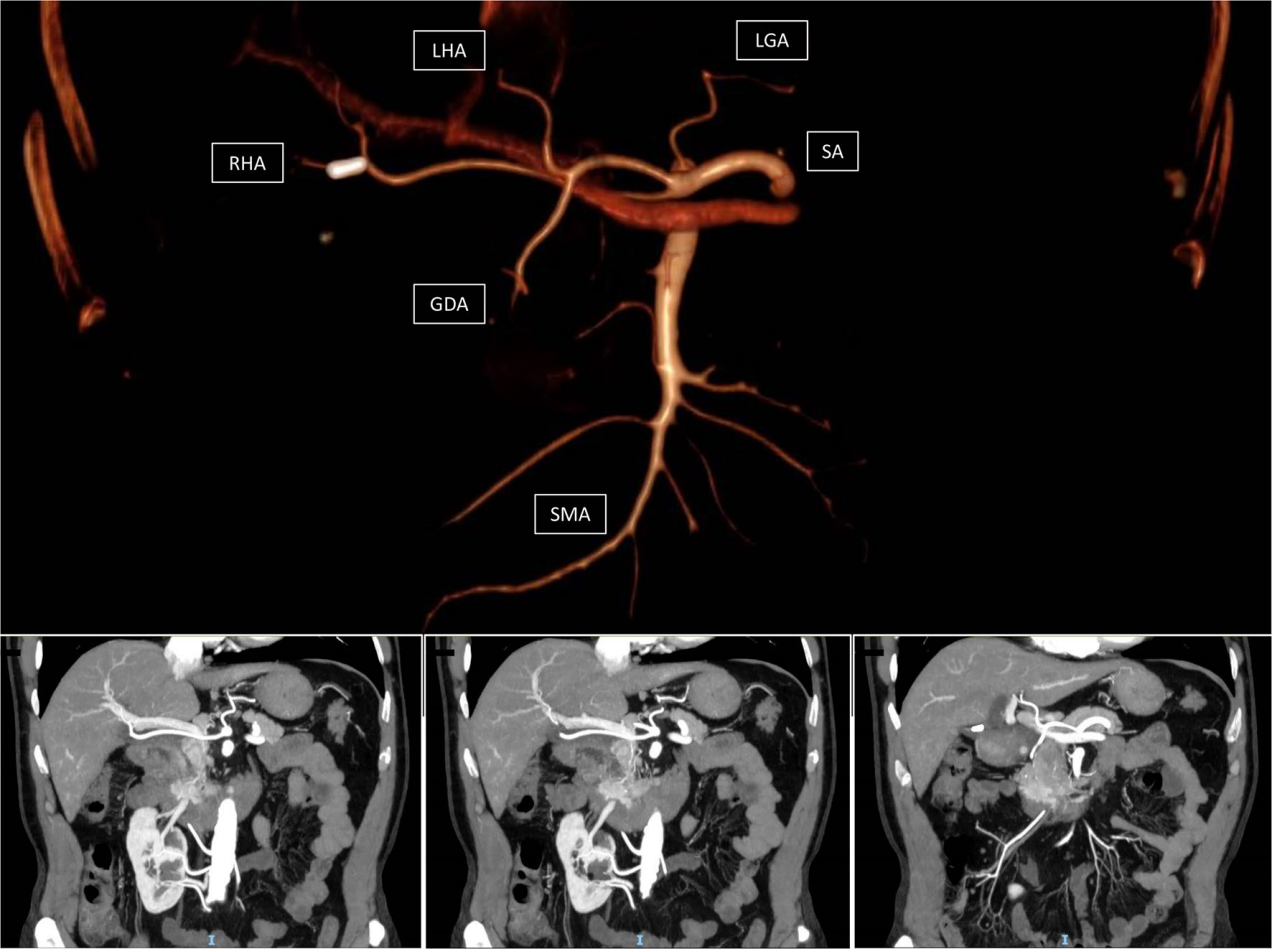
Absence of common and proper hepatic artery. The right and left hepatic arteries (RHA and LHA) arise separately from the celiac trunk (CA). The gastro-duodenal artery (GDA) originates from the left hepatic artery (LHA)

The involvement of an a-RHA in peri-ampullary cancer could imply a neoadjuvant approach because a tumor with a-RHA infiltration or contact should be regarded as technically resectable but oncologically borderline-resectable [45]. In our series, the rate of neoadjuvant therapy was two times higher in patients with a-RHA than in patients with RHA normal anatomy. The correction of this bias is crucial to correctly analyze the R1 resection rate. Indeed, it is well known that the R0 resection rate increases when the neoadjuvant approach is performed [46, 47]. In the present study, the increased R1 resection rate in a-RHA was also confirmed after bias correction, and for this reason, it can be considered robust. In our series, no arterial resection was performed because our group prefers to perform the arterial divestment instead of the arterial resection [48]. The local recurrence rate, DFS, and OS results did not confirm a worse prognosis for patients with a-RHA. However, it should be noted that this analysis was restricted only to the patients resected for PDAC, reducing the sample size of survival analysis (Fig. 4).

Since other factors could influence OS and DFS, the sample size of the present study was too small to guarantee a definitive conclusion.

**Fig. 5:**
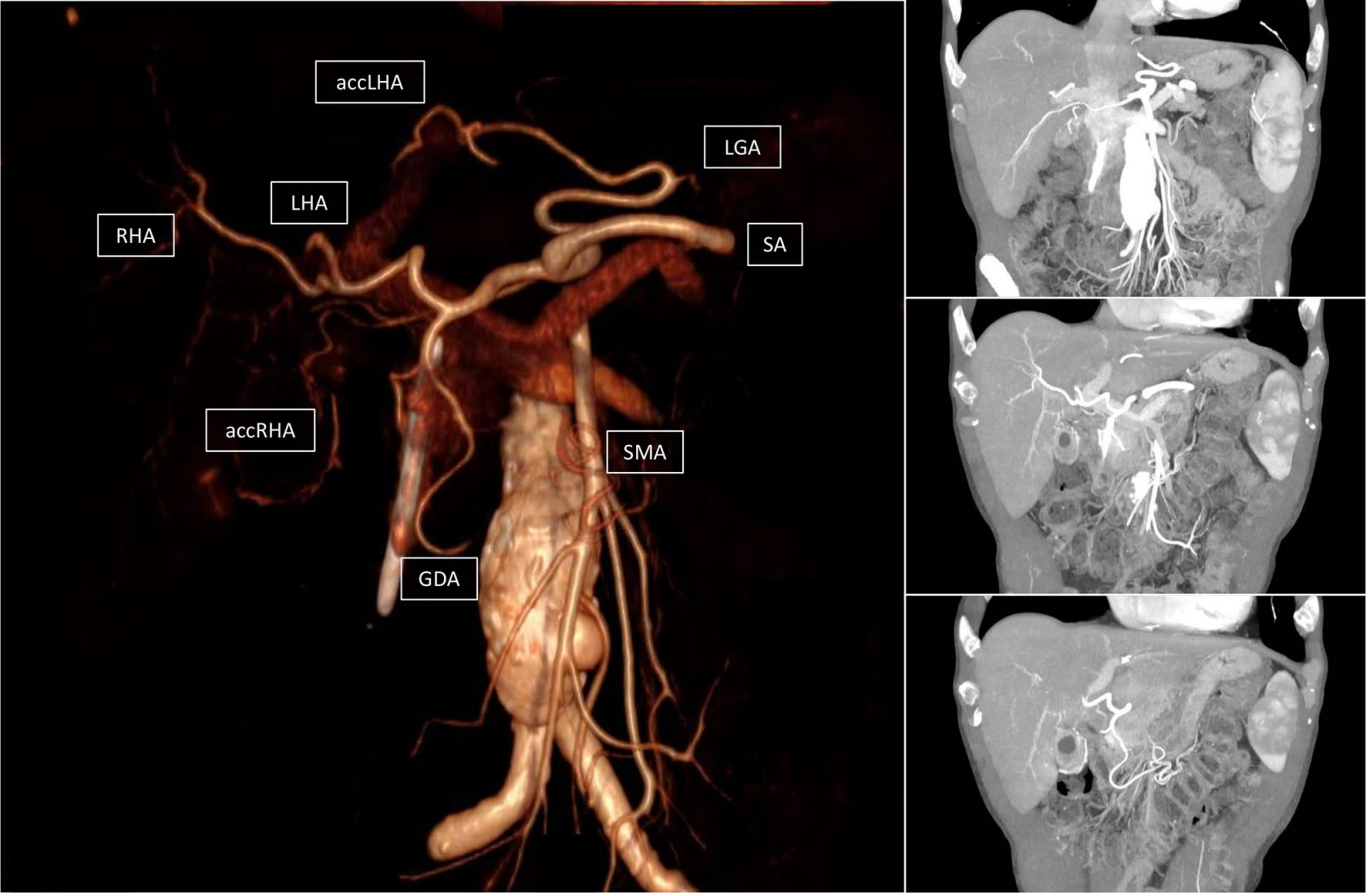
Other anomalies. Co-existence of normal right and left hepatic arteries (right and left hepatic arteries) from common hepatic artery (CHA), accessory right hepatic artery (accRHA) from celiac axis (CA), and accessory left hepatic artery (accLHA) from left gastric artery (LGA)

Another interesting topic is the influence of a-RHA on postoperative morbidity. Ligation or damage of the a-RHA may lead to ischemia of the bile duct with a consecutive biliary leakage of the hepaticojejunostomy, liver abscess, or, rarely, hepatic failure [9]. In the present series, the biliary fistula rate was similar in the two groups without significant differences. This observation did not surprise us because the rate of a-RHA sacrificed or damaged was nil in the present series. Finally, as expected, both groups have similar POPF, DGE, or PPH. Indeed, it is well known that POPF results from several factors, such as pancreatic texture, Wirsung’s size, or BMI of patients [16]. The presence of an a-RHA did not influence the characteristics of the patients or pancreatic remnants related to the POPF. Similarly, complications such as PPH and DGE are due to POPF occurrence rather than damage or ligation of a-RHA. Operative time and LOS were similar between the two groups. Indeed, several factors related to the surgeon's experience or the health care system could influence operative time and LOS. The presence of a-RHA, per se, did not change the operative time or the duration of the postoperative course (Fig. 5).

This study has strengths and limitations. The main strength was that this was the first study in which all specimen margins were analyzed separately. The main limitation was the retrospective design. However, data were retrieved from a prospectively maintained database at a high-volume center. Moreover, entropy balancing analysis reduced the risk of selection bias due to retrospective design. A second limitation was the small sample size, which weakened the conclusion about OS and DFS. A further limitation was that the paper covered a relatively extended time frame during which changes in patient management, such as chemotherapy regimens, occurred.

In conclusion, the present study demonstrated that the presence of an a-RHA could increase the R1 resection rate on arterial margins. This margin was never evaluated alone previously. Further larger and prospective studies should be designed to confirm if a-RHA increases the risk of R1 resection of arterial margins, reducing OS and DFS.

## Supplementary Information

Below is the link to the electronic supplementary material.

Supplementary file1 Figure S1. Flow-chart of patients’ selection process (DOCX 17488 KB)

Supplementary file2 Figure S2. Survival curves in matched and unmatched populations: panel A, OS in un-matched population; panel B, OS in matched population; panel C, DFS in un-matchedpopulation; panel D, DFS in matched population (DOCX 35160 KB)

Supplementary file3 (DOCX 16 KB)

## Data Availability

Data supporting the findings of this study are available from the corresponding author upon reasonable request.

## Abbreviations

PD: Pancreaticoduodenectomy
CT: Computed tomography
a-RHA: Aberrant right hepatic artery
acc-RHA: Accessory right hepatic artery
rep-RHA: Replaced right hepatic artery
CA: Celiac axis
CHA: Common hepatic artery
RHA: Right hepatic artery
LHA: Left hepatic artery
SMA: Superior mesenteric artery
LGA: Left gastric artery
GDA: Gastro-duodenal artery
SMV: Superior mesenteric vein
PV: Portal vein
CDC: Clavien–Dindo classification
CR-POPF: Clinically relevant postoperative pancreatic fistula
PPH: Post-pancreatectomy hemorrhage
DGE: Delayed gastric emptying
LOS: Length of stay
NCCN: National Comprehensive Cancer Network
