# Supplementary figures and images for: Surgical and oncological implications of the presence of hepatic artery anatomical variations in patients undergoing pancreaticoduodenectomy: a single center experience
Source: Updates Surg. 2025 Jan 29;77(2):511–21. doi: 10.1007/s13304-025-02079-3 (PMC11961471; doi:10.1007/s13304-025-02079-3)

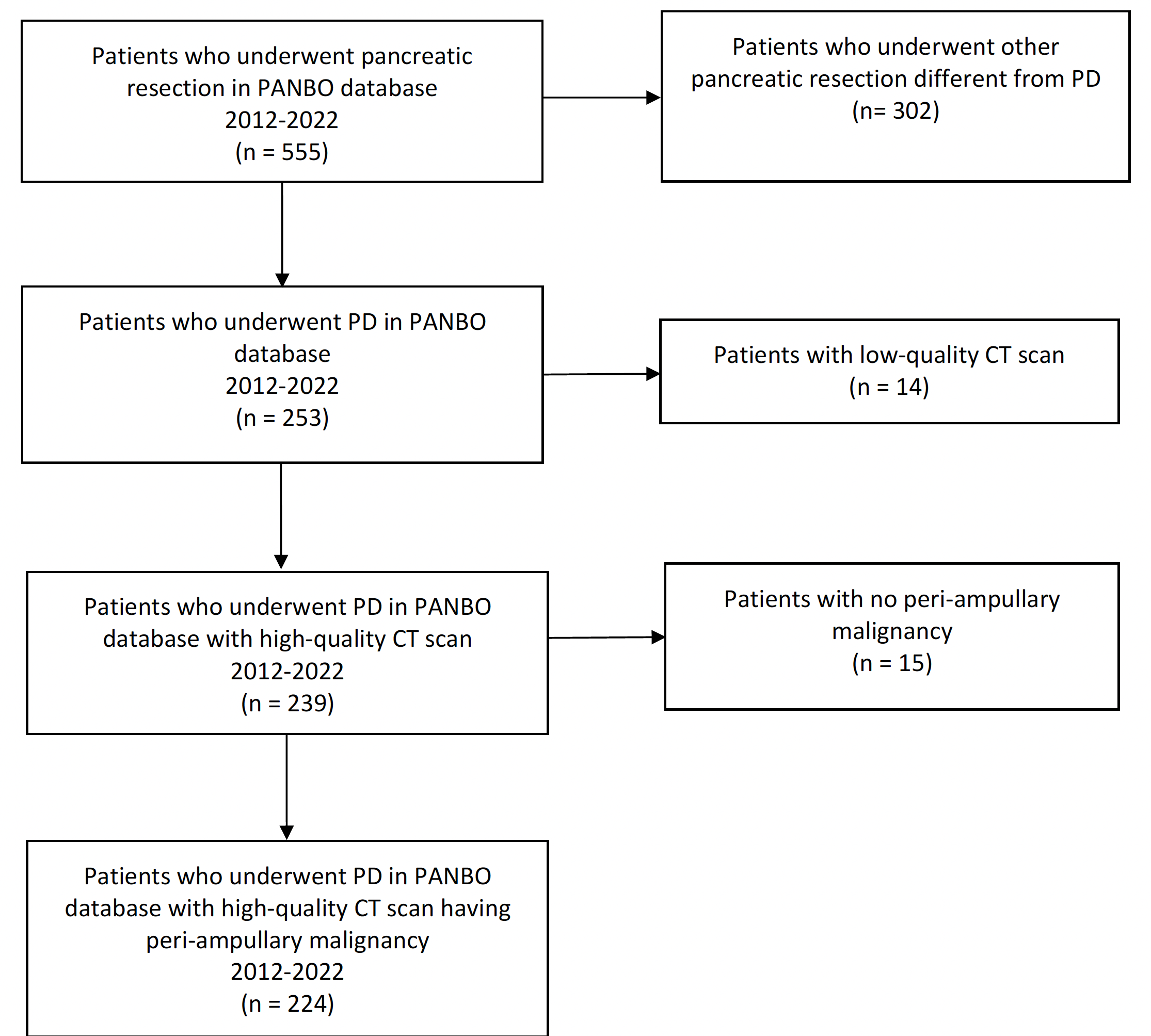

Supplement: Supplementary file 1 — Supplementary file1 Figure S1. Flow-chart of patients’ selection process (DOCX 17488 KB) [file 13304_2025_2079_MOESM1_ESM.tiff]

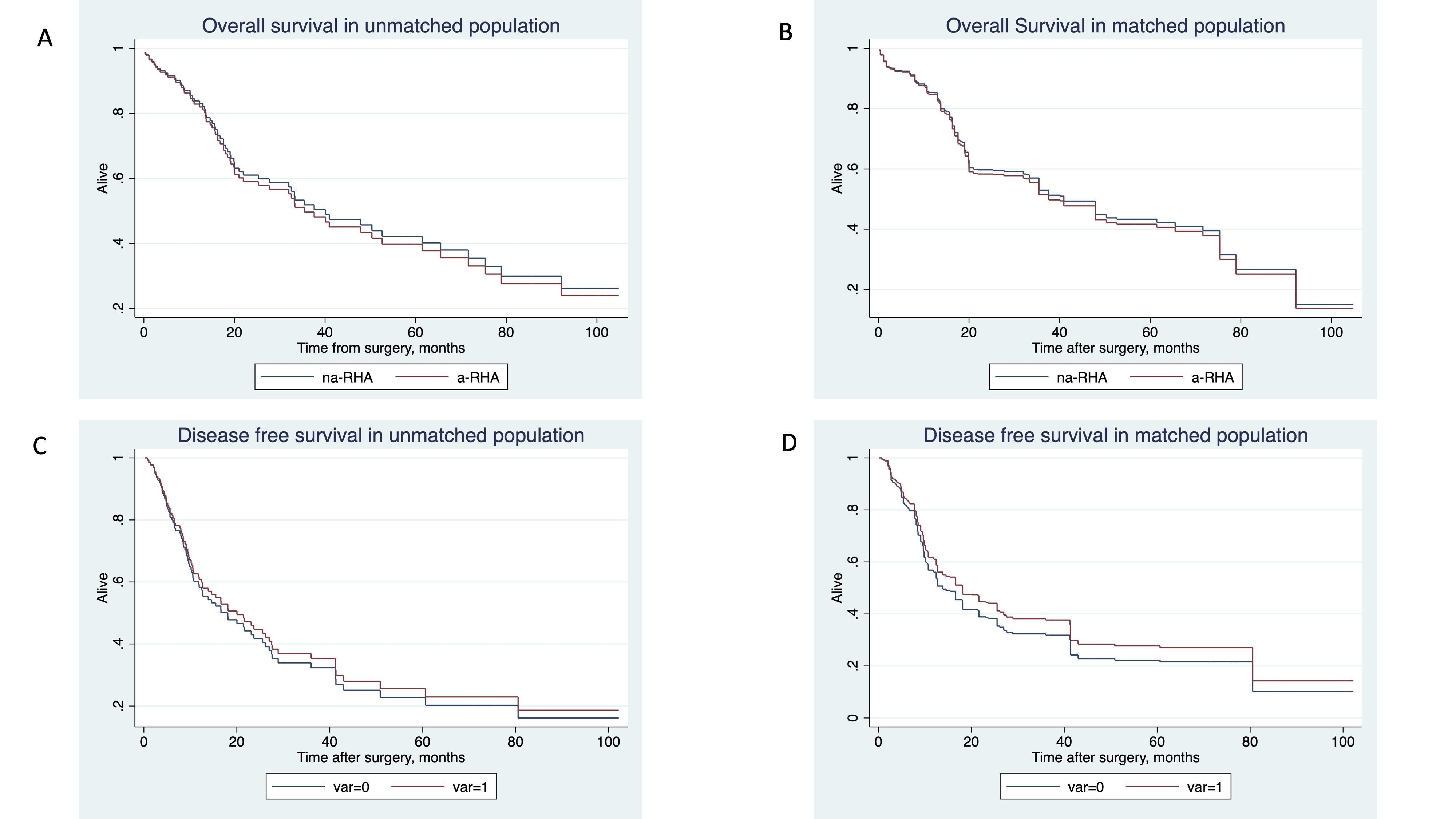

Supplement: Supplementary file 2 — Supplementary file2 Figure S2. Survival curves in matched and unmatched populations: panel A, OS in un-matched population; panel B, OS in matched population; panel C, DFS in un-matchedpopulation; panel D, DFS in matched population (DOCX 35160 KB) [file 13304_2025_2079_MOESM2_ESM.tiff]
