# Supplementary material for: Surgical and oncological implications of the presence of hepatic artery anatomical variations in patients undergoing pancreaticoduodenectomy: a single center experience
Source: Updates Surg. 2025 Jan 29;77(2):511–21. doi: 10.1007/s13304-025-02079-3 (PMC11961471; doi:10.1007/s13304-025-02079-3)
Supplement: Supplementary file 3 — Supplementary file3 (DOCX 16 KB) [file 13304_2025_2079_MOESM3_ESM.docx]

| **Arterial variants according to Michels’ classification** | **Description** | **N. of patients (%)** |
| --- | --- | --- |
|  |  |  |
| Type I | Normal anatomy | 140 (62.5) |
| Type II | Replaced LHA from LGA | 12 (5.4) |
| Type III | Replaced RHA from SMA | 21 (9.4) |
| Type IV | Replaced RHA and Replaced LHA | 1 (0.5) |
| Type V | Accessory LHA from LGA | 25 (11.2) |
| Type VI | Accessory RHA from SMA | 3 (1.3) |
| Type VII | Accessory RHA and Accessory LHA | 3 (1.3) |
| Type VIII | Replaced RHA and Accessory LHA or Replaced LHA and Accessory RHA | 6 (2.6) |
| Type IX | CHA from SMA | 5 (2.2) |
| Type X | CHA from LGA |  |
| Other |  | 8 (3.6) |

**Supplementary Table 1- Anomalies of hepatic artery**

**Legend:** LHA= left hepatic artery; LGA= left gastric artery; RHA= right hepatic artery; SMA= superior mesenteric artery; CHA= common hepatic artery.
